# The complexities of decision-making associated with on-demand treatment of hereditary angioedema (HAE) attacks

**DOI:** 10.1186/s13223-024-00903-w

**Published:** 2024-07-25

**Authors:** Stephen D. Betschel, Teresa Caballero, Douglas H. Jones, Hilary J. Longhurst, Michael Manning, Sally van Kooten, Markus Heckmann, Sherry Danese, Ledia Goga, Autumn Ford Burnette

**Affiliations:** 1 Clinical Immunology and Allergy, Department of Medicine, University of Toronto, Toronto, ON Canada; 2 Servicio de Alergia, Hospital Universitario la Paz, CIBERER U754, IdiPAZ Group 44, Madrid, Spain; 3 Rocky Mountain Allergy at Tanner Clinic, Layton, Utah, USA; 4 Department of Medicine, University of Auckland and Auckland City Hospital, Te Toka Tumai, Auckland, New Zealand; 5 Allergy, Asthma & Immunology Associates, Ltd., Internal Medicine, UA College of Medicine-Phoenix, Scottsdale, AZ USA; 6 KalVista Pharmaceuticals, Inc, Cambridge, MA USA; 7 Outcomes Insights, Agoura Hills, CA USA; 8 Division of Allergy and Immunology, Howard University Hospital, Washington, DC USA; 9 Department Division Director Clinical Immunology and Allergy, Staff Clinical Immunologist and Allergist, University of Toronto, St. Michael’s Hospital, 36 Toronto St, Suite 700, Toronto, ON M5C 2C5 Canada

**Keywords:** Hereditary angioedema (HAE), Attacks, On-demand treatment, Decision-making, Burden of treatment, Survey, HAE attack journey

## Abstract

**Background:**

Hereditary angioedema (HAE) is characterized by debilitating attacks of tissue swelling in various locations. While guidelines recommend the importance of early on-demand treatment, recent data indicate that many patients delay or do not treat their attacks.

**Objective:**

This survey aimed to investigate patient behavior and evaluate the key factors that drive on-demand treatment decision-making, as reported by those living with HAE.

**Methods:**

People living with HAE were recruited by the US Hereditary Angioedema Association (HAEA) to complete a 20-minute online survey between September 6, and October 19, 2022.

**Results:**

Respondents included 107 people with HAE, 80% female, 98% adults (≥ 18 years). Attack management included on-demand therapy only (50%, *n* = 53) or prophylaxis with on-demand therapy (50%, *n* = 54). Most patients (63.6%) reported that they did not carry on-demand treatment at all times when away from home. The most common reason for not carrying on-demand treatment when away from home was ‘prefer to treat at home’ (72.1%). Overall, 86% of respondents reported delaying on-demand treatment, despite recognizing the initial onset of an HAE attack and despite 97% of patients agreeing that it is important to recover quickly from an HAE attack. Reasons for non-treatment or treatment delay included ‘the attack is not severe enough to treat’ (91.9% and 88.0%, respectively), ‘cost of treatment’ (31.1% and 40.2%, respectively), anxiety about refilling the prescription for on-demand treatment quickly (31.1% and 37.0%, respectively), the pain (injection or burning) associated with their on-demand treatment (18.9% and 28.3%, respectively), the lack of a suitable/private area to administer on-demand treatment (17.6% and 27.2%, respectively), lack of time to prepare on-demand treatment (16.2% and 16.3%, respectively), and a ‘fear of needles’ (13% and 12.2%, respectively). Survey findings from the patient perspective revealed that when on-demand treatment was delayed, 75% experienced HAE attacks that progressed in severity, and 80% reported longer attack recovery.

**Conclusions:**

Survey results highlight that decision-making regarding on-demand treatment in HAE is more complicated than expected. The burden associated with current parenteral on-demand therapies is often the cause of treatment delay, despite acknowledgment that delays may result in progression of HAE attacks and longer time to recovery.

## Introduction

Hereditary angioedema (HAE) is a genetic disease resulting in deficiency (type I) or dysfunction (type II) in the complement-1 esterase inhibitor (C1-INH) protein and subsequent uncontrolled activation of the kallikrein kinin system [1]. The symptoms of a HAE attack are well recognized and include swelling in the extremities or face, abdominal pain caused by intestinal swelling, and respiratory difficulties as a result of laryngeal and airway angioedema [2]. The frequency and severity of HAE attacks are highly variable between patients and even can vary within a patient, over their lifetime, making each patient’s HAE attack journey highly individualized [3].

A recent prevalence analysis reported 8,904 HAE patients in the United States (US), which aligns with previous prevalence estimates of 1 in 50,000 within the global population (range 1 in 10,000 to 1 in 150,000) [4–7]. The rarity of HAE may contribute to diagnostic delays for many patients, with time to conclusive diagnosis ranging from 2 to 13.5 years [4, 8].

The recent international World Allergy Organization (WAO)/European Academy of Allergy and Clinical Immunology (EAACI) guideline for HAE acknowledges that early diagnosis and effective therapy are critical for the management of this disabling disease [9]. The goal of HAE treatment is to achieve complete disease control and normalize patients’ lives [9]. Current management of HAE includes the use of medications targeting the kallikrein-kinin pathway for on-demand treatment of acute angioedema events (HAE attacks) with the objective of relieving acute symptoms as quickly and completely as possible [10]. As attacks are unpredictable and potentially life-threatening, it is essential that all HAE patients have effective on-demand therapy readily available [9]. Long-term prophylaxis is recommended for some HAE patients to reduce the frequency of attacks. The need for long-term prophylaxis is highly individualized based on attack frequency and severity, and patient preference [11].

Guidelines state that HAE attacks should be treated early with intravenous (IV) plasma-derived C1-INH and recombinant human C1-INH, plasma kallikrein inhibitor ecallantide administered subcutaneously by a health professional, or self-injected subcutaneous (SC) bradykinin B2 receptor antagonist icatibant, and that early treatment provides far better clinical outcomes than late treatment [11].

For long-term prophylactic treatment, guidelines recommend the use of IV or SC plasma-derived C1-INH, the SC plasma kallikrein monoclonal antibody lanadelumab, or orally administered plasma kallikrein inhibitor berotralstat. Guidelines also recommend an individualized approach to prophylaxis, including careful and regular monitoring of HAE patients who use long-term prophylactic treatment in order to inform dose optimization, with the goal of improving tolerability, adherence to therapy, and quality of life [2, 11].

It is recognized that current HAE treatments are effective and have greatly improved the lives of patients with HAE. Despite these benefits, treatment administration may impose additional burden to some patients and caregivers, as most prophylactic and all on-demand therapies are administered by SC or IV injection [12–14]. Patients have indicated that the route of administration is the factor they consider the most important in their choice of HAE treatment, citing the need for less ”traumatic” administration with oral therapies preferred over SC and IV [13].

Availability of self-administered on-demand parenteral treatments has improved health-related quality of life and HAE attack management. However, deciding when to initiate on-demand therapy may be challenging for patients as attack symptoms may escalate over hours and attacks can be unpredictable and vary in intensity. Patient education is crucial for appropriate and early initiation of on-demand therapy and to minimize attack severity and duration.

However, decision-making associated with on-demand treatment administration as reported by patients and the impact of this process on the patient’s HAE attack journey have not been fully described in the literature. Characterizing patient behavior and understanding the patient perspective are essential for addressing the factors restricting the optimal use of on-demand treatment for HAE attacks. Patients’ experiences, including the self-reported reasons for delaying treatment or not treating attacks, would offer valuable insights into the decision-making process surrounding on-demand treatment administration.

Here, we describe the findings of a patient survey that aimed to encompass the overall behaviors and decision-making process regarding the on-demand treatment of HAE attacks. We present an evaluation of the factors that drive the on-demand treatment decision-making process, including reasons to delay or not treat an attack, as reported by those living with HAE.

## Methods

### Survey development

We conducted an observational, online survey designed to characterize the behaviors and perspectives of people in the US with HAE. The survey questions were original in nature and developed based on a review of the literature (including existing measures), patient focus groups, and clinician expert input. The structure of survey questions included multiple-choice questions with answer choices from a list of provided options (including “Other”), rank-order questions (order of importance 1–5), and scale-based responses using a symmetrical 5-point Likert scale of agreement, with presented statements (from “strongly disagree” to “strongly agree”) consistent with established self-reporting survey methodology [15], and an 11-point Likert scale to evaluate anxiety. An initial pilot survey was developed and then refined through collaboration with patient focus groups, and expert clinicians provided further input for the final survey.

### Participants

Patients completed the 47-question online survey between September 6, 2022 and October 19, 2022. Participants were recruited by the US Hereditary Angioedema Association (HAEA) and were eligible if they were US residents with self-reported, clinician-diagnosed HAE type I or II, and had experienced 1 or more HAE attacks in their lifetime. There were no exclusionary parameters; however, the survey was designed to include an even distribution of respondents based on HAE therapy use (on-demand therapy only or combination of long-term prophylaxis and on-demand therapy). Prior to survey initiation, all materials were reviewed, and a waiver was granted by Advarra, an independent review board. All participants provided informed consent upon entry to the online survey for their data to be used anonymously or in aggregate prior to entering the survey. Participants completed the survey online via a secure web portal/electronic data capture system that took approximately 20 min to complete.

### Statistical analysis

Statistical analysis was performed using Microsoft Excel (Microsoft, Redmond, WA, USA). The analysis plan specified continuous variables to be summarized as means, medians, and ranges, and categorical variables as frequency distributions and percentages. We performed a factor analysis to reduce the number of variables (from 10 individual response options to 3 macro-categories) and then reported associations between the variables using Pearson correlation coefficients.

## Results

### Characteristics of respondents

Of the 155 initial respondents, 107 individuals with HAE within the US completed the survey (69% completion rate). Respondents were predominantly female (80%). The average age was 41 ± 14.6 years (mean ± SD) and 98% of respondents were adults (≥ 18 years). At the time of survey completion, 50% (*n* = 53) of respondents were taking an on-demand therapy only; 50% (*n* = 54) were using a prophylactic treatment in addition to on-demand therapy. Respondent characteristics are presented in Table 1.

**Table 1:** Respondent characteristics

All respondents				
*N* = 107				
**Mean age, years (SD)**	41 (14.6)			
**Age categories, n (%)**				
< 18	2 (1.9%)			
18–24	12 (11.2%)			
25–34	29 (27.1%)			
35–44	22 (20.6%)			
45–54	21 (19.6%)			
55–64	15 (14.0%)			
65–74	4 (3.7%)			
75+	2 (1.9%)			
**Gender, female, n (%)**	86 (80.4%)			
**Treatment type, n (%)**				
Prophylaxis + on-demand	54 (50.5%)			
On-demand only	53 (49.5%)			
**Current prophylactic treatment, n (%)**				
Lanadelumab	31 (29.0%)			
Berotralstat	7 (6.5%)			
C1 esterase inhibitor (subcutaneous)	7 (6.5%)			
Androgens/steroids	5 (4.7%)			
C1 esterase inhibitor (intravenous)	4 (3.7%)			
Not taking prophylactic treatment	53 (49.5%)			
**Current primary on-demand treatment, n (%)**				
Icatibant	84 (78.5%)			
C1 esterase inhibitor (recombinant)	13 (12.1%)			
C1 esterase inhibitor (human)	9 (8.4%)			
Ecallantide	1 (0.9%)			
All respondents *N* = 107				
**Mean age, years (SD)**			41 (14.6)	
**Age categories, n (%)** < 18 18–24 25–34 35–44 45–54 55–64 65–74 75+			2 (1.9%) 12 (11.2%) 29 (27.1%) 22 (20.6%) 21 (19.6%) 15 (14.0%) 4 (3.7%) 2 (1.9%)	
**Gender, female, n (%)**			86 (80.4%)	
**Treatment type, n (%)** Prophylaxis + on-demand On-demand only			54 (50.5%) 53 (49.5%)	
**Current prophylactic treatment, n (%)**				
Lanadelumab Berotralstat C1 esterase inhibitor (subcutaneous) Androgens/steroids C1 esterase inhibitor (intravenous) Not taking prophylactic treatment			31 (29.0%) 7 (6.5%) 7 (6.5%) 5 (4.7%) 4 (3.7%) 53 (49.5%)	
**Current primary on-demand treatment, n (%)** Icatibant C1 esterase inhibitor (recombinant) C1 esterase inhibitor (human) Ecallantide			84 (78.5%) 13 (12.1%) 9 (8.4%) 1 (0.9%)	

SD, standard deviation

### Recognition of the health consequences of treatment and non-treatment of an HAE attack

Three-quarters (75%) of respondents reported that when on-demand treatment was delayed, their HAE attacks were more severe and 80% stated that recovery from their attacks took notably longer. These findings were consistent amongst those on prophylaxis (74.1% and 79.6%) and those using on-demand treatment only (75.5% and 81.1%). The vast majority of respondents also recognized that the decision not to treat an HAE attack impacted their plans for the remainder of the day; 89% stated their plans for the day change if they do not treat an HAE attack. This was similar for those on prophylaxis (96%) and those currently using on-demand treatment only (82%). Almost all respondents (95%) reported that they experienced a decreased level of anxiety once they realized they were recovering from the attack and nearly all (97%) agreed that it is important to recover quickly from an HAE attack.

### Delayed treatment or non-treatment of HAE attacks

Despite their appreciation of the consequences of non-treatment, 63.6% of patients reported that they did not carry an HAE on-demand treatment at all times when away from home. The most common reason for not carrying on-demand treatment when away from home was ‘prefer to treat at home’ (72.1%). More than half (57%) of respondents reported that they do not treat all attacks (64% of on-demand only patients; 50% of those on prophylaxis). On average, one of every five (20%) HAE attacks are untreated (24% amongst on-demand only patients; 15% amongst prophylaxis patients).

14% reported that they immediately treat all HAE attacks (9.4% of on-demand only patients; 18.5% of those on prophylaxis). Even though respondents were able to recognize the initial onset of an attack, the majority (86%) chose to delay on-demand treatment administration.

**Fig. 1:**
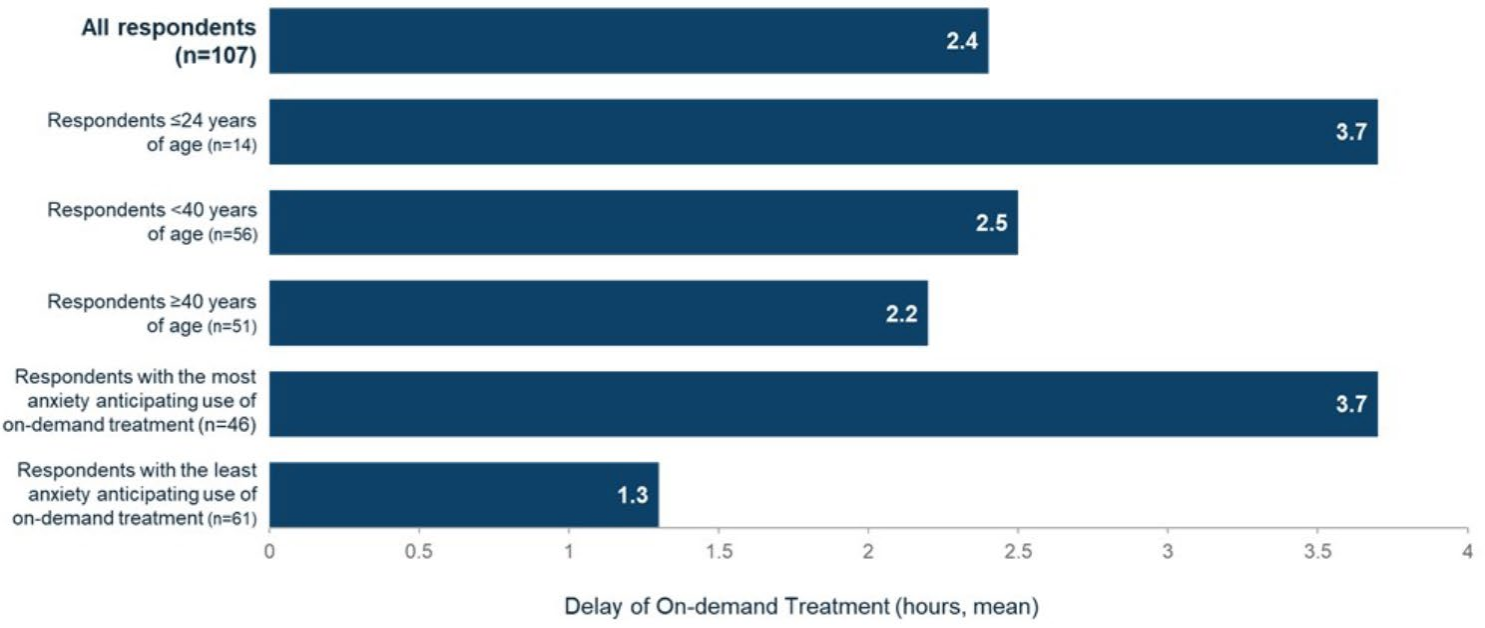
Duration of delay in administration of on-demand treatment after initial recognition of HAE attack onset

On average, respondents reported waiting an average of 2.4 h to treat their HAE attack after recognizing the initial onset of the attack (Fig. 1). Younger people (≤ 24 years of age, 13% of the cohort, *n* = 14) reported waiting on average 3.7 h to administer on-demand treatment. The majority of HAE patients in this survey were taking icatibant as their primary on-demand treatment (78.5%, *n* = 84). Compared with patients who used other on-demand treatments (C1 inhibitors or ecallantide), patients taking icatibant delayed administration the longest (2.6 h [mean] vs. 1.3 h [mean]). The time to treatment with icatibant was consistent amongst those on prophylaxis (2.0-hour delay, mean) and those using on-demand treatment only (2.7-hour delay, mean).

Respondents that delayed treatment were less likely to treat all of their attacks. Patients who waited one hour or more to treat their HAE attack after initial recognition of the attack onset (57%) did not treat 27.4% of their attacks. In comparison, those that typically treated their attacks within an hour of recognizing the attack onset were more likely to treat all of their attacks, with only 9.7% of attacks going untreated.

**Fig. 2:**
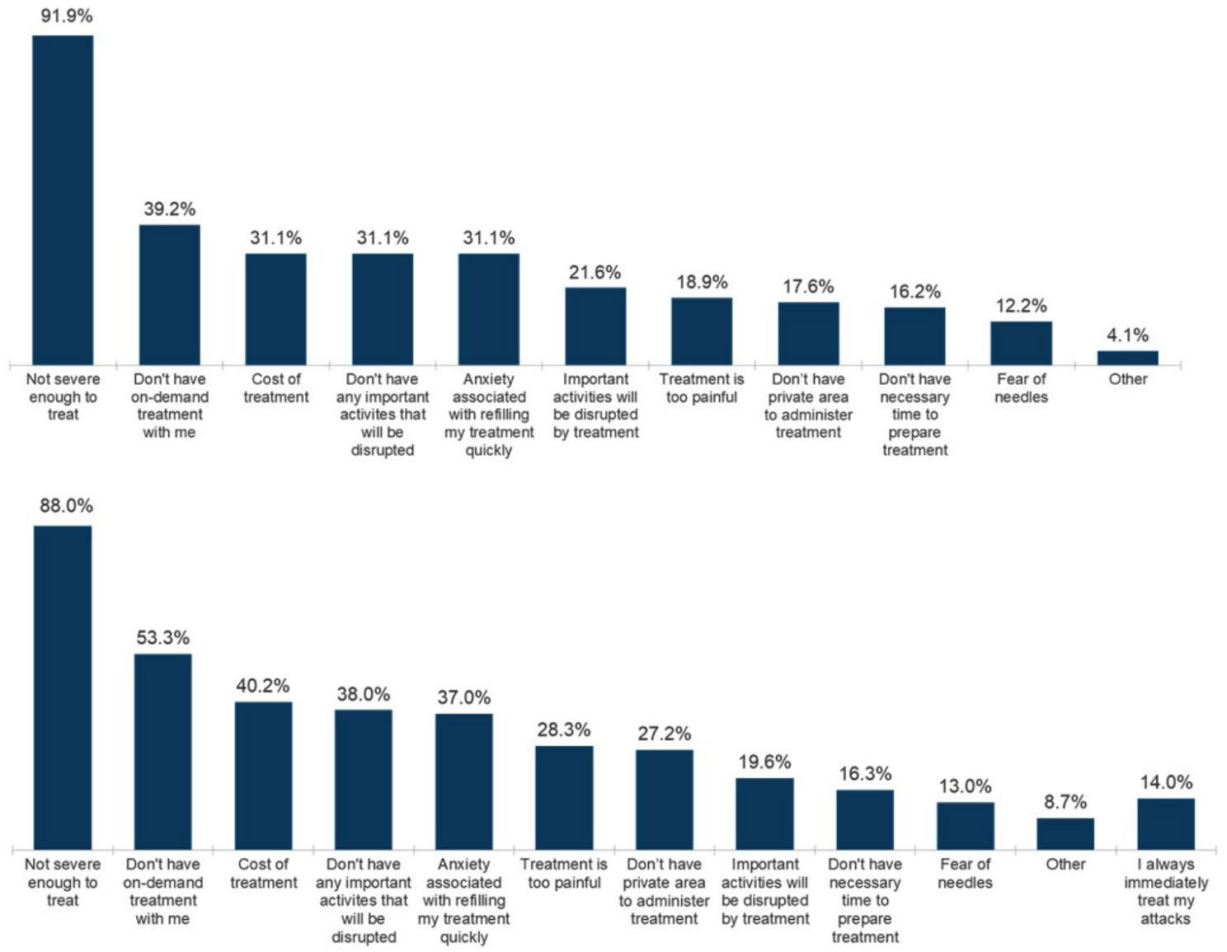
**A** Reasons provided for not treating an HAE attack (*n* = 74, excluding those who treat all of their attacks, *n* = 33) – respondents could select as many as they liked **B** Reasons provided for waiting to treat an HAE attack at recognition of attack onset (*n* = 92, excluding those that immediately treat their HAE attacks, *n* = 15) – respondents could select as many as they liked

Within their survey responses, people with HAE reported reasons for treatment delay or non-treatment. Responses are summarized in Fig. 2 A/B. The single most common reason respondents do not treat (91.9%) or delay treating an attack (88.0%) is because they question if the attack is severe enough to treat. Notably, many of the HAE patients do not treat an attack (39.2%) or delay treating an attack (53.3%) because they do not have their on-demand treatment with them at the time of symptom onset. Other reported reasons for non-treatment or treatment delay included cost of treatment (31.1% and 40.2%, respectively), anxiety about refilling the prescription for on-demand treatment quickly (31.1% and 37.0%, respectively), the pain (injection or burning) associated with their on-demand treatment (18.9% and 28.3%, respectively), the lack of a suitable/private area to administer on-demand treatment (17.6% and 27.2%, respectively), lack of time to prepare on-demand treatment (16.2% and 16.3%, respectively), and a fear of needles (13.0% and 12.2%, respectively). Individuals currently on prophylaxis reported that they were more anxious about refilling their prescription for on-demand treatment quickly (43%) than those using on-demand only (23%), which contributed to the decision to not treat the attack.

### Anxiety associated with anticipating on-demand treatment

Anxiety was shown to be a key contributing factor to delayed administration of on-demand treatment. Respondents reported a mean of 4.2 on a numeric rating scale of 0 to 10 (“not anxious,” [0]; “mildly anxious,” [1–3]; “moderately anxious,” [4–6]; “extremely anxious,” [7–10]) when anticipating the use of their current on-demand treatment. Those who reported feeling moderately to extremely anxious (rating of 4 or higher) when they anticipated the use of current on-demand treatment (49% of total) also reported that they delayed treatment, waiting 1 to 2 h (median). Approximately two thirds (65%) of those with HAE who were more anxious about on-demand treatment, also do not treat all their attacks. Similar percentages of patients felt anxious anticipating the use of on-demand treatment regardless of prophylaxis use, with 83% of those who were on prophylaxis reported to be anxious anticipating the use of on-demand treatment as compared with 81% of those who used on-demand treatment only.

Factor analysis demonstrated correlations between the following paired response variables:

- ‘The attack is not severe enough to treat’ AND ‘I do not have on-demand treatment with me’ (Pearson correlation coefficient 0.39).
- ‘I have a fear of needles’ AND ‘My on-demand treatment is too painful’ (Pearson correlation coefficient 0.35).

## Discussion/Conclusions

While major advances have been made with the availability of effective parenteral on-demand treatment options and the development of prophylactic treatments for HAE, patients including those on prophylaxis can still experience HAE attacks requiring on-demand therapy. Thus, the WAO/EAACI guideline states that all patients should have and carry on-demand medication for the treatment of at least two HAE attacks [9]. The findings of this patient survey characterize a personal decision-making process for why, when, and where to administer currently available on-demand treatment despite early recognition of attacks by patients and clear understanding of the benefits of early treatment.

While self-administration of current parenteral on-demand therapies has improved opportunities for HAE attack management, the decisions faced by patients about if and when to administer treatment may be complex. The survey found that despite recognizing the initial onset of an attack, many patients delay administration of on-demand treatment while also acknowledging that delayed treatment leads to a longer recovery time. Respondents provided various reasons for doing so, with most respondents answering that the attack is not severe enough. This response is also linked directly to patients feeling the need to justify giving themselves permission to treat as a result of evaluating many other burden of treatment parameters such as, ‘treatment is too painful,’ ‘lack of a suitable/private area to administer treatment,’ and, ‘fear of needles,’ as key factors that influence their treatment decisions. The results showing the reasons for delaying or forgoing on-demand treatment highlight the need for patient and physician education regarding the importance of shared decision-making on the prompt recognition and appropriate treatment of HAE attacks. In addition, access to on-demand treatment weighs on the minds of patients and causes anxiety when they consider the cost of treatment and ability to get refills quickly. Survey results indicated patients using prophylaxis also use on-demand therapy more often than on-demand only patients. One explanation might be that patients in the former group may have a higher level of comfort and experience with medications and injections than those in the latter group. Regardless of prophylaxis status, the financial burden associated with HAE medications is well documented and may play a significant role in the decision to treat for all patients, with high treatment costs serving as a barrier to access [16]. The WAO/EAACI guideline states that all attacks should be treated as early as possible [9] yet this survey demonstrated that patients felt the need to justify that an attack was worth treating due to many different reasons, some of which are not captured explicitly in this survey, and that are independent of the severity or intensity of attack symptoms.

The survey results highlight that there are complexities associated with the decision to treat. Most patients (89%) stated that their decision to treat or not influenced their plans for the day, with possible consequences for work or school activities as well as economic and social implications. Despite recognizing the consequences, patients report delaying or forgoing on-demand treatment even with the availability of effective parenteral treatment. These findings emphasize the need to address the reasons why patients choose to delay or forgo on-demand treatment even with the knowledge of the potential for serious outcomes. Therefore, it is critical for clinicians to be aware of patients’ perspectives, which allows for a greater understanding of their motivations, behaviors, and barriers to on-demand treatment adherence.

Given the inherent nature of survey-related research, it is important to acknowledge that the results presented here be interpreted considering the following limitations. Due to the anonymity of survey responses, verification of self-reported data is not possible. Additionally, reported baseline anxiety and increased anxiety are based on patient reports and an accurate determination of the degree to which each attack contributed to overall anxiety is not feasible. We also acknowledge that factors such as disease duration, frequency of attacks, time since last attack and experience with individual therapies may have influenced the reported outcomes. Future studies are needed to determine the extent of these influences on patient responses. Although this would be considered a large sample for a rare disease, all findings may not represent a broader or more global population. The majority of survey respondents were female (80.4%), indicating a higher proportion of females compared with previous research which cited 60% female predominance [17]. This finding is consistent with previous research showing higher online survey response rates among females compared with males [18] and may also reflect the greater severity of HAE observed in females [17]. The current survey is also subject to potential sampling bias due to it being available online only and administered in English, limiting the potential to reach certain populations including non-English speakers and those with limited access to the internet. Participants were recruited by the HAEA, which has a deep and established relationship with the US HAE population, and these patients may likely be more educated on HAE than the general HAE patient population. These findings were observational only and not reported in comparison with any control group. Although the survey questions used were original in nature and customized to the study objectives, the survey itself was not derived from existing validated instruments.

We acknowledge that patient-driven surveys are subjective in nature; however, they do present the patients’ experience, which may help to identify gaps in care. Analyzing patient behavior by exploring patients’ perceptions regarding HAE treatment is vital for facilitating informed decision-making and optimizing HAE management. This is the first characterization of the on-demand treatment decision-making process that people living with HAE undergo as part of the HAE attack journey. This survey also highlights the how characteristics of current parenteral on-demand treatments contribute to suboptimal attack management by patients. Incorporating the patient perspective into care strategies can lead to more patient-centered approaches that enhance treatment adherence and improve overall outcomes. These findings may help to inform future treatment discussions and may enhance aspects of the physician/patient dialogue, aiding in building patient behaviors that are aligned with current guidelines and goals of treatment [19].

## Data Availability

Due to the rarity of the disease, it may be possible to link anonymized patient data back to individual patients. Therefore, only aggregate data will be shared. Inquiries from qualified researchers should be sent to: DSP@kalvista.com.
