# Correction: The complexities of decision-making associated with on-demand treatment of hereditary angioedema (HAE) attacks

**DOI:** 10.1186/s13223-025-00960-9

**Published:** 2025-04-07

**Authors:** Stephen D. Betschel, Teresa Caballero, Douglas H. Jones, Hilary J. Longhurst, Michael Manning, Sally van Kooten, Markus Heckmann, Sherry Danese, Ledia Goga, Autumn Ford Burnette

**Affiliations:** 1 Clinical Immunology and Allergy, Department of Medicine, University of Toronto, Toronto, ON Canada; 2 Servicio de Alergia, Hospital Universitario la Paz, CIBERER U754, IdiPAZ Group 44, Madrid, Spain; 3 Rocky Mountain Allergy at Tanner Clinic, Layton, Utah, USA; 4 Department of Medicine, University of Auckland and Auckland City Hospital, Te Toka Tumai, Auckland, New Zealand; 5 Allergy, Asthma & Immunology Associates, Ltd., Internal Medicine, UA College of Medicine-Phoenix, Scottsdale, AZ USA; 6 KalVista Pharmaceuticals, Inc, Cambridge, MA USA; 7 Outcomes Insights, Agoura Hills, CA USA; 8 Division of Allergy and Immunology, Howard University Hospital, Washington, DC USA; 9 Department Division Director Clinical Immunology and Allergy, Staff Clinical Immunologist and Allergist, University of Toronto, St. Michael’s Hospital, 36 Toronto St, Suite 700, Toronto, ON M5C 2C5 Canada

**Correction to: Allergy, Asthma & Clinical Immunology (2024) 20:43**

10.1186/s13223-024-00903-w

In this article [1], the author would like to switch the order of a couple of words that refer to data so as to accurately align with Fig. 2A and B.

In the section ‘Results’, the text “Other reported reasons for non-treatment or treatment delay included cost of treatment (31.1% and 40.2%, respectively), anxiety about refilling the prescription for on-demand treatment quickly (31.1% and 37.0%, respectively), the pain (injection or burning) associated with their on-demand treatment (18.9% and 28.3%, respectively), the lack of a suitable/private area to administer on-demand treatment (17.6% and 27.2%, respectively), lack of time to prepare on-demand treatment (16.2% and 16.3%, respectively), and a fear of needles (13.0% and 12.2%, respectively).”

Should read as

Other reported reasons for non-treatment or treatment delay included cost of treatment (31.1% and 40.2%, respectively), anxiety about refilling the prescription for on-demand treatment quickly (31.1% and 37.0%, respectively), the pain (injection or burning) associated with their on-demand treatment (18.9% and 28.3%, respectively), the lack of a suitable/private area to administer on-demand treatment (17.6% and 27.2%, respectively), lack of time to prepare on-demand treatment (16.2% and 16.3%, respectively), and a fear of needles (12.2% and 13.0%, respectively).
